# Modulation of Drug Resistance in* Staphylococcus aureus* with Coumarin Derivatives

**DOI:** 10.1155/2016/6894758

**Published:** 2016-04-19

**Authors:** Rodrigo Santos Aquino de Araújo, José Maria Barbosa-Filho, Marcus Tullius Scotti, Luciana Scotti, Ryldene Marques Duarte da Cruz, Vivyanne dos Santos Falcão-Silva, José Pinto de Siqueira-Júnior, Francisco Jaime Bezerra Mendonça-Junior

**Affiliations:** ^1^Post-Graduate Program in Natural and Synthetic Bioactive Products, Federal University of Paraíba, 58051-900 João Pessoa, PB, Brazil; ^2^Laboratory of Genetics of Microorganisms, Molecular Biology Department, Federal University of Paraíba, 58051-900 João Pessoa, PB, Brazil; ^3^Laboratory of Synthesis and Drug Delivery, Biological Science Department, State University of Paraíba, 58071-160 João Pessoa, PB, Brazil

## Abstract

Semisynthetic and commercial coumarins were investigated for their antibacterial and adjuvant properties with antibiotic agents against norfloxacin, erythromycin, and tetracycline resistant* Staphylococcus aureus* as based on efflux mechanisms. The coumarins and certain commercial antibiotics had their Minimum Inhibitory Concentrations determined by broth microdilution assay against resistant* S. aureus* strains which overexpress efflux pump proteins. For evaluation of the modulatory activity, the antibiotics MICs were determined in the presence of the coumarin derivatives at subinhibitory concentration. Although the coumarins did not display relevant antibacterial activity (MIC ≥ 128 *µ*g/mL), they did modulate the antibiotics activities. Various coumarins, especially the alkylated derivatives in combination with antibiotics at subinhibitory concentrations, modulated antibiotic activity, reducing the MIC for tetracycline and norfloxacin by 2 to 8 times. Polar Surface Area (PSA) studies were performed and the fact that the presence of apolar groups is an important factor for the modulatory activity of coumarins was corroborated. Docking on the Penicillin-Binding Protein from MRSA identified that** 18** is a potential ligand presenting low *E*_binding_. The results indicate that coumarin derivatives modulated antibiotic resistance and may be used as potential antibiotic adjuvants, acting by bacterial efflux pump inhibition in* S. aureus*.

## 1. Introduction

The development of mechanisms of resistance by the microorganisms is a natural process that occurs in greater intensity with the indiscriminate and inappropriate use of antimicrobials [1]. This natural adaptation phenomenon has become the principle problem worldwide for the treatment of infectious diseases, creating a continuous need for the discovery of new chemical entities with antibiotic properties [2].

Bacterial infections are a major cause of morbidity and mortality in hospitals, as in the case of nosocomial infections caused by* Staphylococcus aureus*, which cause about 12,000 deaths per year in the USA [3]. It is mentioned in the literature that* S. aureus* is a versatile pathogen with numerous virulence factors, making this microorganism capable of acquiring antibiotic resistance determinants [4]. Resistance to methicillin (MRSA) first appeared in 1961 [5], with currently described* S. aureus* strains having acquired resistance to all *β*-lactams, macrolides, aminoglycosides, tetracycline, rifampicin, quinolones, and even vancomycin [6].

Among the various mechanisms of bacterial resistance, transmembrane efflux proteins have been commonly associated with resistance to multiple antibiotics and other chemotherapeutic agents [7, 8]. These proteins actively extrude toxic substrates, including antibiotics, from the cell. Antibiotic activity-modifying agents or modulators are compounds that potentiate the activity of antibiotics against resistant strains; some of these agents act as efflux pump inhibitors (EPI) [9]. Some efflux pumps have been shown to be of clinical significance in bacterial infections [10], among which are the TetK pump (promoting resistance to tetracycline), the MsrA macrolide efflux protein (promoting macrolide resistance), and the NorA efflux pump (with resistance to fluoroquinolones) [11].

Currently, one of the best strategies to control bacterial resistance and extend the life of existing antibiotics is to associate them with modulators of drug resistance, similar to what is done with many *β*-lactam antibiotics that are administered in combination with potassium clavulanate. Compounds that potentiate the activity of antimicrobial agents present numerous benefits including the possibility of reducing the antibiotic concentration in prophylactic or therapeutic treatments of infection and reversion of cell resistance to conventional therapy.

Coumarins comprise a class of natural phenolic compounds characterized by a single benzene fused to an *α*-pyrone ring. They stand out for having great biological potential, as demonstrated in several studies, compounds with antifungal [12] and antibacterial activities [13], and also modulators of antibiotic resistance [14].

Given the above, and knowing the antimicrobial potential and modulating action of coumarin derivatives on antibiotic resistance, our goal was to develop semisynthetic coumarin derivatives in order to evaluate their antibiotic and/or antibiotic adjuvant properties against effluxing* S. aureus* strains.

## 2. Materials and Methods

### 2.1. Coumarin Derivatives and Antibiotics

All commercial coumarins (1,2-Benzopyrone** 1**; 3-Hydroxycoumarin** 2**; 4-Hydroxycoumarin** 3**; 6-Hydroxycoumarin** 4**; 7-Hydroxycoumarin** 5**; 6,7-Dihydroxycoumarin** 6**; Coumarin-3-carboxylic acid** 7**; 3,3′-Methylene-bis-(4-hydroxycoumarin)** 8**; 6-Methoxy-7-hydroxycoumarin** 9**; and 7,8-Dihydroxy-6-methoxycoumarin** 10**), reagents, and solvents were purchased from Sigma-Aldrich (Seelze, Germany) and used without further purification. Norfloxacin, erythromycin, and tetracycline were obtained from Sigma Chemical Co., USA, and their stock solutions were then prepared [15]. Compounds** 11**–**22** were synthesized according to previously reported procedures [12]. The stock solutions of coumarin derivatives** 1**–**22** were prepared in DMSO. The highest concentration remaining after broth dilution (4%) did not inhibit bacterial growth, and a positive control with only DMSO 4% was tested and showed no interference with bacterial growth (data not shown).

### 2.2. Bacterial Strains

Four* S. aureus* strains were used for the biological tests: one standard strain (ATCC 6538) and three antibiotics resistant strains: SA-1199B, which over expresses the* norA* gene encoding the NorA efflux protein [16]; RN4220 harboring plasmid pUL5054, which carries the gene encoding the MsrA macrolide efflux protein [17]; and IS-58, which possesses the TetK tetracycline efflux protein [18]. The strains, kindly provided by Professor Simon Gibbons (University of London), were maintained in blood agar base (Laboratórios Difco Ltda., Brazil) slants. Prior to use, the cells were grown overnight at 37°C in brain heart infusion broth (BHI-Laboratórios Difco Ltda., Brazil).

### 2.3. Drug Susceptibility Testing and Modulation Assay

The Minimum Inhibitory Concentrations (MICs) of the antibiotics and coumarin derivatives were determined in BHI by broth microdilution assay using a suspension of ca. 10^5^ cfu/mL and a drug concentration range from 0.5 to 256 *µ*g/mL (twofold serial dilutions). For better visualization of the bacterial growth (after 24 h) we used resazurin (0.01%). To evaluate coumarin as a modulator of drug resistance “modulation assay,” we used a method that has been widely applied to identify potential EPIs and is valid provided that one uses specifically known effluxing strains. The MICs of the antibiotics were determined in the presence of the subinhibitory concentrations of coumarin compounds. Both assays were performed in triplicate [19, 20].

### 2.4. Molecular Model

Three-dimensional structures were drawn using HyperChem 8.0 software [21]; the structures had energy-minimization calculated employing the method MM+ force field without any restriction [22]. Subsequently, a new geometry optimization process, based on the semiempirical method AM1 (Austin Model 1) was performed [23]. The optimized structures were subjected to conformation analysis using the random search method with 1,000 interactions, 100 cycles of optimization, and 10 lowest minimum energy conformers. The selected dihedrals were evaluated for rotation in accordance with the standard (default) conditions of the program; the number of simultaneous variations was 1 to 8. Acyclic chains were submitted to rotations from 60 to 180°; torsion rings were in the range of 30 to 120° [24, 25]. The lowest energy conformers were selected and imported into VEJA ZZ. The drawn coumarins were imported into the VEJA ZZ program [26] for calculation of the* Polar Surface Area* (PSA).

### 2.5. Docking

The structure of the Penicillin-Binding Protein (PBP) from Methicillin Resistant* Staphylococcus aureus* (MRSA) in complex with the ligand quinazolinone (PDB id: 4CJN) [27] was downloaded from the Protein Data Bank (http://www.rcsb.org/pdb/home/home.do). Coumarin derivatives** 11**,** 13**, and** 16**–**19** were submitted to molecular docking using the Molegro Virtual Docker, v. 6.0.1 (MVD) [28].

The protein and compound structures were prepared using the default parameter settings in the software package (score function: MolDock Score; ligand evaluation: Internal ES, Internal H-Bond, and sp^2^–sp^2^ Torsions, all checked; number of runs: 10 runs; algorithm: MolDock SE; maximum interactions: 1500; max. population size: 50; max. steps: 300; neighbor distance factor: 1.00; max. number of poses returned: 5). The docking procedure was performed using a GRID of 15 Ǻ in radius and 0.30 in resolution to cover the ligand-biding site of the Penicillin-Binding Protein (PBP) structure.

Templates with features expected to be relevant for ligand binding (quinazoline) were generated to perform docking. The MolDock Score [GRID] algorithm was used as the score function, and the MolDock search algorithm was used [28].

## 3. Results and Discussion

**Scheme 1:**
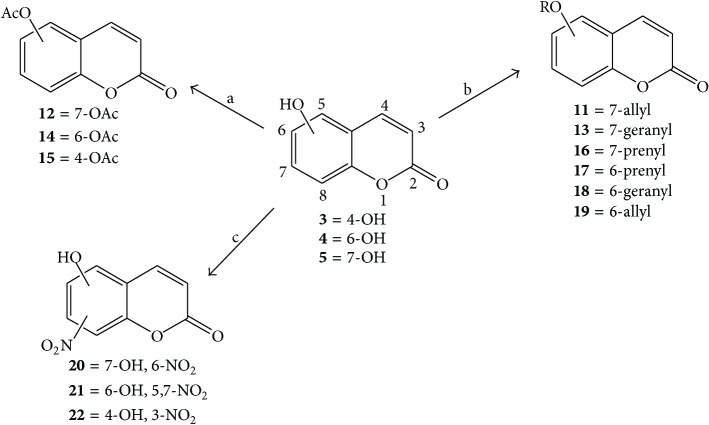
Synthesis of alkyl-, acetyl-, and nitro-coumarin derivatives. Reagents and conditions: (a) acetic anhydride, pyridine, rt, ultrasound irradiation; (b) allyl bromide, geranyl bromide, or prenyl bromide, K_2_CO_3_, acetonitrile, and reflux; (c) HNO_3_/AcOH, 0–5°C for 30 min and then 90 min at rt.

Twelve coumarin derivatives (**11**–**22**) were synthesized from commercial coumarins 4-hydroxy- (**3**), 6-hydroxy- (**4**), and 7-hydroxycoumarin (**5**) by alkylation, acetylation, and nitration procedures according to procedures previously described by our group [12] (Scheme 1).

All commercial (**1**–**10**) and synthetic (**11**–**22**) coumarins were evaluated for their* in vitro* antibacterial activity against the four* Staphylococcus aureus* strains: the standard (ATCC 6538) and the three resistant: IS-58 (TetK), RN4220 (MsrA), and SA-1199B (NorA); however, all of the commercial coumarins (**1**–**10**) and the eight synthetic coumarins (**11**,** 12**,** 15**, and** 18**–**22**) showed no antibacterial activity (MIC > 256 *µ*g/mL) against any* S. aureus* strain evaluated. Compounds** 13**,** 14**,** 16**, and** 17** showed weak activity with MIC values of 128 *µ*g/mL (**13** against ATCC 6538 and SA-1199B,** 16** against SA-1199B, and** 14** and** 17** against all strains).

Shakeel-U-Rehman and colleagues [29] demonstrated antibacterial activity of coumarin compounds (with a free hydroxyl at position 8) against resistant and susceptible strains of* S. aureus* and also reported reduction in activity with acetylation of coumarin precursors. This came into disagreement with our acetylated compound (**14**). Disagreement with our results was also seen in comparison with the studies of Céspedes et al. [30], who reported good antibacterial activities for compounds with dihydroxyls at positions C-7 and C-8 and methoxylation at C-6 against susceptible strains of* S. aureus*, unlike our compounds** 6** and** 10**, which revealed no antibacterial activity against strains of this microorganism.

In the assay for bacterial resistance (modulation assay) the MICs of the antibiotics (tetracycline against IS-58 strain, erythromycin against RN4220 strain, and norfloxacin against SA-1199B strain) were determined in the absence and in the presence of the coumarin derivatives (compounds** 1**–**22**) which were incorporated into the growth medium at a concentration corresponding to 1/4 of the MIC (subinhibitory concentration) [19]. The results of the compounds that showed modulatory activity are presented in Table 1.

As can be seen in Table 1, some coumarins enhanced the activity of tetracycline (2x) and of norfloxacin (2x to 8x), thus reducing the concentration required to inhibit the growth of drug resistant (effluxing) strains.

Among the commercial coumarins evaluated only 1,2-Benzopyrone (**1**) showed modulatory activity, reducing the MIC of norfloxacin in twice. However, the synthesized compounds having allyl (**11**,** 19**), geranyl (**13**,** 18**), and prenyl (**16** and** 17**) radicals showed good to significant modulatory activities, in particular for resistance to norfloxacin (Table 1).

The common characteristic of these modulatory compounds is the presence of an alkyl radical, which has been described as important for the drug resistance modulating activity of* S. aureus*, inhibiting the efflux system [31]. Brunel et al. [32] have also associated the presence of the geranyl group as an important characteristic for modulation of multiple drug resistance in Gram-negative bacteria.

Changing the position of these C-7 radicals (**11**,** 13**, and** 16**) to the C-6 position (**17**,** 18**, and** 19**) favored the modulation of the tetracycline resistance by two times but reduced (two to four times) the modifying activity of norfloxacin. With respect to the modulatory activity of coumarin for the RN4220 (MRSA) strain, no reduction was observed for the erythromycin MIC. However, Reynolds et al. [33] have suggested that the MRSA protein cannot be an efflux pump protein.

Acetylated and nitrated coumarins were all inactive, which leads us to believe that the presence of electron withdrawing groups (decreasing the electron density of the benzopyran ring) or polar radicals negatively contribute to both antibiotic and modulatory activities for this class of compounds.

These conclusions based on a preliminary SAR study were also proven by analyzing the calculations of the Polar Surfaces Area (PSA) of the compounds, obtained by the VEJA ZZ program [26]. It was observed that the absence of free hydroxyls, carboxylic acids, and other polar groups (i.e., nitro derivatives) seems to be an important factor for the modulatory activity of coumarins.

**Figure 1:**
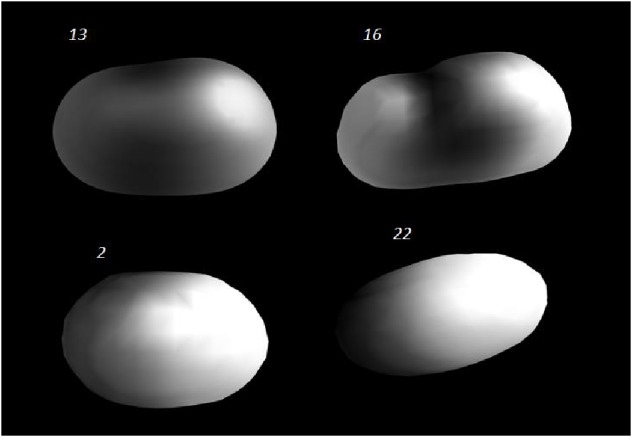
Representation of the PSA (Polar Surface Area) of active (**13** and** 16**) and inactive (**2** and** 22**) compounds using* VEJA ZZ* program. White represents polar surface and grey-dark represents no-polar surfaces.

Figure 1 shows the performed PSA, which allows observing the compounds that have polar surfaces (black) (**2** and** 22**) and which are less active than the nonpolar derivatives (grey) (**13** and** 16**).

**Figure 2:**
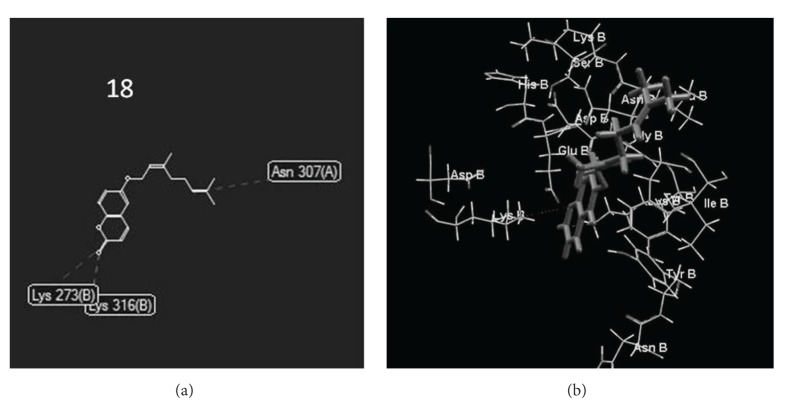
Interactions observed in the compound** 18**-PBP complex.

Additionally, docking study of the most active coumarins (**11**,** 13**, and** 16**–**19**) against the Penicillin-Binding Protein (PBP) from Methicillin Resistant* Staphylococcus aureus* (MRSA) (PDB id: 4CJN) was performed.  Table 2 shows the results of the MolDock Score of these potential inhibitors, and Figure 2 shows the interactions observed in the complex of the best ligand (compound** 18**) with the active site of the PBP enzyme.

One of bacterial resistance pathways occurs by low-interaction of the antibiotic by PPBs and hydrolysis by *β*-lactamases. These bacterial proteins are fundamental in the synthesis of the cell wall of bacteria, common target of *β*-lactam antibiotics. This fact makes PPBs a target searched against a resistance, particularly in Gram-positive bacteria like* Staphylococcus aureus* and also in a higher selectivity to these unique bacteria structures [34–38].

Molecular docking can be formulated as an optimization problem, where the task is to find the ligand-binding mode with the lowest energy.  Table 2 shows the energies (kcal/mol) obtained from the interaction of the coumarins** 11**,** 13**, and** 16**–**19** and quinazolinone (compound complex with the enzyme in the crystal structure) and the PPB. We observed that compounds** 17** (*E*_binding_ = −6224 kcal/mol) and** 18** (*E*_binding_ = –91322 kcal/mol) generated lower energy scores which represent better protein-ligand bindings and stable complexes.

Compound** 18** has shown greater energy value than the quinazolinone (*E*_binding_ = −71098 kcal/mol) ligand complexed in the PDB. Figure 2 reports in 2D (a) and in 3D (b) the interactions formed between** 18** and PPB: we can see hydrogen bond with LYS316 of chain B and two steric bonds (ASN307 of chain A and LYS273 of chain B).

## 4. Conclusions

Acting as antibiotic adjuvants, synthetic coumarin derivatives, especially alkylated derivatives, have shown promising antibiotic activity. They act by modulating antibiotic resistance through a mechanism that most likely involves inhibition of bacterial efflux pumps, thus affecting* S. aureus* resistance to tetracycline and norfloxacin.

The preliminary SAR study was confirmed by PSA analysis and reinforces the idea that coumarin derivatives substituted with electron donors groups are more powerful. These findings may help future design and synthesis of new derivatives, with more potent modulatory activity.

The docking study performed against the Penicillin-Binding Protein (PBP) from Methicillin Resistant* Staphylococcus aureus* (MRSA) identified that compound** 18** is the best ligand with lower energy scores being one potential inhibitor of this important enzyme.

Considering the natural and inevitable development of resistance mechanisms in microorganisms, especially* S. aureus*, and the constant need for new agents with antibiotic properties, our results contribute to the problematic of bacterial infections. The discovery of new chemical entities associable with conventional antibiotics (antibiotic adjuvants) may allow reversion of cell resistance to conventional therapy (increased useful antibiotic life) and thus reduce antibiotic concentrations in prophylactic or therapeutic treatments.

## Figures and Tables

**Table 1:** MIC values (*μ*g/mL) of antibiotics in the absence and presence of coumarin derivatives against *S. aureus* strains: SA-1199B (NorA), RN4220 (MsrA), and IS-58 (TetK).

Antibiotic + coumarin	MIC (*μ*g/mL)		
	IS-58 (TetK) Tetracycline	RN4220 (MrsA) Erythromycin	SA-1199B (NorA) Norfloxacin
Antibiotics alone	64	>256	128
Antibiotics + **1**	64	>256	64 (2x)
Antibiotics + **11**	64	>256	32 (4x)
Antibiotics + **13**	64	>256	32 (4x)
Antibiotics + **16**	64	>256	16 (8x)
Antibiotics + **17**	32 (2x)	>256	64 (2x)
Antibiotics + **18**	32 (2x)	>256	64 (2x)
Antibiotics + **19**	64	>256	64 (2x)

**Table 2:** Binding energy (*E*_binding_) values.

Compounds	MolDock Score (kcal/mol)
Quinazolinone	−71098
**11**	−45453
**13**	−58358
**16**	−58021
**17**	−62224
**18**	−91322
**19**	−55676

## References

[1] French G. L. (2010). The continuing crisis in antibiotic resistance. *International Journal of Antimicrobial Agents*.

[2] Jordheim L. P., Ben Larbi S., Fendrich O. (2012). Gemcitabine is active against clinical multiresistant *Staphylococcus aureus* strains and is synergistic with gentamicin. *International Journal of Antimicrobial Agents*.

[3] Noskin G. A., Rubin R. J., Schentag J. J. (2005). The burden of *Staphylococcus aureus* infections on hospitals in the United States: an analysis of the 2000 and 2001 Nationwide Inpatient Sample Database. *Archives of Internal Medicine*.

[4] Chambers H. F., DeLeo F. R. (2009). Waves of resistance: *Staphylococcus aureus* in the antibiotic era. *Nature Reviews Microbiology*.

[5] Fang Y.-H., Hsueh P.-R., Hu J.-J. (2004). Community-acquired methicillin-resistant *Staphylococcus aureus* in children in northern Taiwan. *Journal of Microbiology, Immunology and Infection*.

[6] Hiramatsu K., Katayama Y., Yuzawa H., Ito T. (2002). Molecular genetics of methicillin-resistant *Staphylococcus aureus*. *International Journal of Medical Microbiology*.

[7] Marquez B. (2005). Bacterial efflux systems and efflux pumps inhibitors. *Biochimie*.

[8] Piddock L. J. V. (2006). Clinically relevant chromosomally encoded multidrug resistance efflux pumps in bacteria. *Clinical Microbiology Reviews*.

[9] Gibbons S. (2004). Anti-staphylococcal plant natural products. *Natural Product Reports*.

[10] Li X.-Z., Nikaido H. (2004). Efflux-mediated drug resistance in bacteria. *Drugs*.

[11] Van Bambeke F., Pagès J.-M., Lee V. J. (2006). Inhibitors of bacterial efflux pumps as adjuvants in antibiotic treatments and diagnostic tools for detection of resistance by efflux. *Frontiers in Anti-Infective Drug Discovery*.

[12] de Araújo R. S. A., Guerra F. Q. S., Lima E. D. O. (2013). Synthesis, Structure-activity Relationships (SAR) and *in silico* studies of coumarin derivatives with antifungal activity. *International Journal of Molecular Sciences*.

[13] Hamdi N., Al-Ayed A. S., Said R. B., Fabienne A. (2012). Synthesis and characterization of new thiazolidinones containing coumarin moieties and their antibacterial and antioxidant activities. *Molecules*.

[14] Bazzaz B. S. F., Iranshahi M., Naderinasab M., Hajian S., Sabeti Z., Masumi E. (2010). Evaluation of the effects of galbanic acid from *Ferula szowitsiana* and conferol from *F. badrakema*, as modulators of multi-drug resistance in clinical isolates of *Escherichia coli* and *Staphylococcus aureus*. *Research in Pharmaceutical Sciences*.

[15] CLSI—Clinical and Laboratory Standards Institute (2015). Performance standards for antimicrobial susceptibility testing; twenty-fifth informational supplement. *CLSI Document*.

[16] Kaatz G. W., Seo S. M. (1995). Inducible NorA-mediated multidrug resistance in *Staphylococcus aureus*. *Antimicrobial Agents and Chemotherapy*.

[17] Ross J. I., Farrell A. M., Eady E. A., Cove J. H., Cunliffe W. J. (1989). Characterisation and molecular cloning of the novel macrolide-streptogramin B resistance determinant from *Staphylococcus epidermidis*. *Journal of Antimicrobial Chemotherapy*.

[18] Gibbons S., Udo E. E. (2000). The effect of reserpine, a modulator of multidrug efflux pumps, on the *in vitro* activity of tetracycline against clinical isolates of methicillin resistant *Staphylococcus aureus* (MRSA) possessing the *tet*(K) determinant. *Phytotherapy Research*.

[19] Stavri M., Piddock L. J. V., Gibbons S. (2007). Bacterial efflux pump inhibitors from natural sources. *Journal of Antimicrobial Chemotherapy*.

[20] Falcão-Silva V. S., Silva D. A., Souza M. D. F. V., Siqueira J. P. (2009). Modulation of drug resistance in *Staphylococcus aureus* by a kaempferol glycoside from *Herissantia tiubae* (Malvaceae). *Phytotherapy Research*.

[22] Allinger N. L. (1977). Conformational analysis. 130. MM2. A hydrocarbon force field utilizing V1 and V2 torsional terms. *Journal of the American Chemical Society*.

[23] Dewar M. J. S., Zoebisch G., Healy E. F., Stewart J. J. P. (1985). The development and use of quantum-mechanical molecular-models. 76. AM1—a new general-purpose quantum-mechanical molecular model. *Journal of the American Chemical Society*.

[24] Cohen N. C. (1996). *Guidebook on Molecular Modeling in Drug Design*.

[25] Leach A. R. (2001). *Molecular Modeling: Principles and Applications*.

[26] Pedretti A., Villa L., Vistoli G. (2004). VEGA-an open platform to develop chemo-bio-informatics applications, using plug-in architecture and script programming. *Journal of Computer-Aided Molecular Design*.

[27] Bouley R., Kumarasiri M., Peng Z. (2015). Discovery of antibiotic (*E*)-3-(3-carboxyphenyl)-2-(4-cyanostyryl)quinazolin-4(3*H*)-one. *Journal of the American Chemical Society*.

[28] Thomsen R., Christensen M. H. (2006). MolDock: a new technique for high-accuracy molecular docking. *Journal of Medicinal Chemistry*.

[29] Shakeel-U-Rehman, Khan R., Bhat K. A., Raja A. F., Shawl A. S., Alam M. S. (2010). Isolation, characterisation and antibacterial activity studies of coumarins from *Rhododendron lepidotum* Wall. ex G. Don, Ericaceae. *Brazilian Journal of Pharmacognosy*.

[30] Céspedes C. L., Avila J. G., Martínez A., Serrato B., Calderón-Mugica J. C., Salgado-Garciglia R. (2006). Antifungal and antibacterial activities of Mexican tarragon (*Tagetes lucida*). *Journal of Agricultural and Food Chemistry*.

[31] Kumar A., Khan I. A., Koul S. (2008). Novel structural analogues of piperidine as inhibitors of the NorA efflux pump of *Staphylococcus aureus*. *Journal of Antimicrobial Chemotherapy*.

[32] Brunel J. M., Lieutaud A., Lome V., Pagès J.-M., Bolla J.-M. (2013). Polyamino geranic derivatives as new chemosensitizers to combat antibiotic resistant Gram-negative bacteria. *Bioorganic & Medicinal Chemistry*.

[33] Reynolds E., Ross J. I., Cove J. H. (2003). Msr(A) and related macrolide/streptogramin resistance determinants: incomplete transporters?. *International Journal of Antimicrobial Agents*.

[34] Zervosen A., Sauvage E., Frère J.-M., Charlier P., Luxen A. (2012). Development of new drugs for an old target—the penicillin binding proteins. *Molecules*.

[35] Fernandes M. B., Gonçalves J. E., Scotti M. T., de Oliveira A. A., Tavares L. C., Storpirtis S. (2012). Caco-2 cells cytotoxicity of nifuroxazide derivatives with potential activity against Methicillin-resistant *Staphylococcus aureus* (MRSA). *Toxicology in Vitro*.

[36] Greninger A. L., Chatterjee S. S., Chan L. C. (2016). Whole-genome sequencing of methicillin-resistant *Staphylococcus aureus* resistant to fifth-generation cephalosporins reveals potential non-meca mechanisms of resistance. *Plos ONE*.

[37] Hong S. B., Rhee M. H., Yun B., Lim Y. H., Song H. G., Shin K. S. (2016). Synergistic anti-bacterial effects of *Phellinus baumii* ethyl acetate extracts and beta-lactam antimicrobial agents against methicillin-resistant *Staphylococcus aureus*. *Annals of Laboratory Medicine*.

[38] Lavanya P., Ramaiah S., Anbarasu A. (2016). A molecular docking and dynamics study to screen potent anti-staphylococcal compounds against ceftaroline resistant MRSA. *Journal of Cellular Biochemistry*.

